# HIV Self-Test Program Preferences Among Non-Hispanic Black and Hispanic Men Who Have Sex with Men in the Southern United States: A Discrete Choice Experiment

**DOI:** 10.1007/s10461-026-05125-2

**Published:** 2026-04-17

**Authors:** John Guigayoma, Jason J. Ong, Mariano Kanamori, DeMarc Hickson, Lori Ward, Sara J. Becker, Katie Biello, Tyler Wray

**Affiliations:** 1 Impact Institute, Northwestern University, Chicago, IL USA; 2 Faculty of Medicine, School of Translational Medicine, Nursing and Health Sciences, Monash University, Melbourne, VIC Australia; 3 Melbourne Sexual Health Centre, Alfred Health, Carlton, VIC Australia; 4 Faculty of Infectious and Tropical Diseases, London School of Hygiene and Tropical Medicine, London, UK; 5 Department of Public Health Sciences, School of Medicine, University of Miami, Miami, FL USA; 6 Us Helping Us, People Into Living Inc, Washington, DC USA; 7 School of Public Health, University of Memphis, Memphis, TN USA; 8 Center for Dissemination and Implementation Science, Northwestern University Feinberg School of Medicine, Chicago, IL USA; 9 Department of Epidemiology, Brown University School of Public Health, Providence, RI USA; 10 Department of Behavioral & Social Sciences, Brown University School of Public Health, Providence, RI USA; 11 The Fenway Institute, Fenway Health, Boston, MA USA

**Keywords:** Men who have sex with men, Southern United States, HIV self-testing, Discrete choice experiment, Black/African American, Hispanic/Latino

## Abstract

Non-Hispanic Black and Hispanic men who have sex with men (BHMSM) in the Southern United States (US) have the highest rates of new HIV diagnoses in the country. Self-testing can increase detection and contribute to decreasing diagnoses, but it is unclear which self-test program characteristics encourage the highest engagement. To inform the design of self-test programs, we conducted an online discrete choice experiment with BHMSM in this region who did not have an HIV diagnosis (*n* = 290) to understand their preferences for the following self-test program attributes: delivery strategy (home delivery, friend delivery, clinic pickup), support (instructions only, during the test, 1 week after delivery), delivery speed (same day, next day, 3 days, 5 days), and price ($0, $20, $40, $50, $60). We analyzed choice data using random parameters to estimate preferences and marginal willingness to pay. BHMSM preferred free (b = 2.67) self-tests, home delivery (b = 0.60), and same-day (b = 0.24) or next-day (b = 0.35) delivery and had a preference against support 1 week after delivery (b=-0.19). Marginal willingness to pay estimates showed that BHMSM would pay $6.71 more for home delivery compared to clinic pickup and $9.10 more for same-day delivery and $10.96 more for next-day delivery compared to 5-day delivery. Based on these results, a self-test program with free rapid home delivery and support via instructions only or during the test would best address the preferences of BHMSM in the Southern US. Patients may also be willing to pay for rapid home delivery, highlighting the possibility of cost-sharing models for self-test programs.

**Supplementary Information:**

The online version contains supplementary material available at https://doi.org/10.1007/s10461-026-05125-2.

## Introduction

In 2022, more than a quarter of new HIV diagnoses in the United States (US) were among non-Hispanic Black and Hispanic men who have sex with men (BHMSM) in the South [1]. HIV self-testing can contribute to reducing new HIV infections by identifying undiagnosed HIV infections before onward transmission can occur [2]. However, a national survey of sexual minority men found that less than 8% had used a self-test in the past year [3], suggesting the need for increased implementation of self-testing programs, especially in high prevalence regions such as the Southern US.

One major implementation barrier for self-testing programs is that it is unclear how programs should be designed to optimize engagement. Self-testing programs use a range of strategies for delivering tests and providing support with linkage to care and prevention services [4–6], yet it is unclear which of these characteristics will encourage self-test use among BHMSM in the Southern US. Information on which program characteristics patients want the most can guide organizations and funders on which program designs to adopt or finance. The limited efforts in other US regions to explore self-test program preferences include two studies that recruited BHMSM in Los Angeles and gave participants the option to receive their tests via home delivery, pharmacy pickup or vending machine [7, 8]. In both studies, most participants chose home delivery, but neither study provided data comparing home delivery to other successful strategies such as social-network delivery [9, 10] or clinic pickup [11]. Research also shows that counseling support during or after testing could be an effective way to link newly diagnosed patients to HIV care [12], but given that privacy is a major advantage of self-testing [13, 14], there is inadequate research on what patients want from a support strategy. In addition, patients may value how quickly they receive tests because of convenience [13, 14], but few trials reported the delivery speeds of their self-test programs [9, 11, 15] and likely used longer delivery times that were most affordable.

Given that public health programs typically provide self-tests for free, another major implementation barrier is the operational costs of these programs [5, 6]. Although government and philanthropic grants typically fund these efforts, relying exclusively on these sources of support can impede widespread implementation. For example, one study of a free national self-test home delivery program reported that for every test mailed to a patient, the implementing organization paid $28 per kit and $15 in shipping and handling [16], which over hundreds of tests delivered are substantial costs. To facilitate implementation of self-testing, community-based organizations and health departments may consider other approaches to offering self-testing sustainably, including potential cost-sharing by patients. However, few studies are available on what BHMSM in the Southern US might be willing to pay for self-tests. Previous research shows that the $40 retail cost of the OraQuick self-test is too high for many patients and that patients greatly prefer free tests [5, 6], but some studies suggest that a substantial subset of patients would be willing to pay $10-$20 for a test [11, 17]. Knowledge of willingness to pay can help organizations plan pricing strategies for their own self-test programs and can help funders account for possible cost-sharing by patients.

To inform the design of self-test programs, we conducted an online discrete choice experiment with BHMSM who live in the Southern US. Discrete choice experiments are survey tools that provide estimates of preferences for program attributes and corresponding marginal willingness to pay for these attributes and can thus inform the planning and implementation of public health programs [18].

## Methods

We have published the protocol for this discrete choice experiment elsewhere [19] and summarize briefly below.

### Recruitment

We embedded recruitment within the online campaigns for two pragmatic trials of sexual minority men who test for HIV infrequently [20, 21] from March 2023 to December 2023. These campaigns consisted of ads on popular social media sites such as Facebook and Instagram and videos delivered by social media influencers with LGBTQ audiences on sites such as YouTube and TikTok [22]. The campaigns were placed in various United States Department of Health and Human Services Ending the HIV Epidemic jurisdictions in the South [23]. Potential participants who clicked the ads completed an online survey to assess eligibility criteria. Those who qualified for this survey were routed to an online informed consent.

### Participants

Eligible participants were: (1) at least 18 years old, (2) identified as cisgender male or transgender, gender nonconforming, or nonbinary, (3) non-Hispanic Black or Hispanic, (4) lived in a state in the Southern US, (5) reported having had anal or vaginal sex with a man in the past year, (6) had not been diagnosed with HIV, and (7) were fluent in English or Spanish. Eligibility was based on screener responses. The 17 Southern states that qualified for this study are based on the US Census Bureau-defined South region [24]: Alabama, Arkansas, Delaware, Florida, Georgia, Kentucky, Louisiana, Maryland, Mississippi, North Carolina, Oklahoma, South Carolina, Tennessee, Texas, Virginia, Washington, DC, and West Virginia. In our screener, we asked questions about race and ethnicity. We categorized those who selected “Black or African American” only for race and “not Hispanic” for ethnicity as “non-Hispanic Black” and classified all those who selected “Hispanic” for ethnicity regardless of any race selected (including Black or African American) as “Hispanic.” We used these categorizations to align with Centers for Disease Control and Prevention categorizations [1].

### Procedures

We built all study materials in English and Spanish on Qualtrics (Qualtrics LLC, Provo, Utah). Survey materials in Spanish were also culturally tailored to the Latino community by a team of Spanish-speaking HIV researchers with extensive experience conducting research with Latino populations. Participants who completed the informed consent were routed to the survey. Those who completed the survey received $25 via a reloadable debit card sent to their homes. All study procedures received approval from the Brown University Institutional Review Board.

### Discrete choice experiment design

Our online survey consisted of a discrete choice experiment to understand participants’ preferences and corresponding marginal willingness to pay for several self-test program attributes. Discrete choice experiments are based on random utility theory [25, 26], which states that consumers choose services that provide the most satisfaction (or “utility”) based on the service’s various characteristics various characteristics (or “attributes”). Discrete choice experiments provide independent preference estimates for each potential characteristic (or “attribute level”) [27]. More specifically, the higher the preference estimate for a potential characteristic, the more important that characteristic is to a consumer, and the more likely consumers would participate in a program with that characteristic in the program’s design. For this discrete choice experiment, we asked participants to consider self-test programs that delivered the OraQuick In-Home HIV Test (OraSure Technologies Inc, Bethlehem, PA) an oral swab test that can detect HIV antibodies in 20 min and that is the only test approved by the US Food and Drug Administration for rapid self-testing [28].

The discrete choice experiment consisted of four attributes: delivery strategy (home delivery, friend delivery, clinic pickup), support (instructions only, while you test, 1 week after delivery), delivery speed (same day, next day, 3 days, 5 days), and price ($0, $20, $40, $50, $60. We used the term “friend delivery” instead of “social-network delivery” to ease interpretation for participants. Attributes were based on scientific literature on the characteristics of successful self-test programs, and attribute levels were based on successful self-test clinical trials and highly feasible program designs along with possible delivery times and prices [19]. For delivery strategy, we chose “home delivery” based on two successful randomized controlled trials [11, 15], “friend delivery” based on another successful trial [9], and “clinic pick-up” based on a highly feasible program design [29].

For support, we based the “instructions only” attribute level on package instructions provided by the manufacturer [30], and we based the “during the test” and “1 week after delivery” levels on support strategies within previous successful trials and common support strategies in existing HIV self-test programs [31], For delivery speed, we based attribute levels on available delivery times in public and private mail delivery services [32, 33]. For price, we based the $0 level on how nearly all publicly funded HIV self-test programs provide tests for free [5, 6] and the $40 level on the typical retail price of the test [34, 35]. We based the $20 level on potential patient cost-sharing off the retail value based on previous research [11, 17] and the $50 and $60 levels based on the possibility that BHMSM may pay more than retail value for tests if programs provide features beyond typical HIV self-test program designs, such as friend delivery, faster delivery times, or support beyond package instructions.

Before completing the discrete choice experiment, participants read survey prompts that describe each attribute and attribute level. Supplementary Material 1 provides these prompts. The descriptions for the delivery strategy attribute (home delivery, friend delivery, clinic pick-up), provided information on how patients would receive an HIV self-test. For “home delivery,” patients would order a test through a local testing clinic’s website and the clinic would mail tests out to patients’ homes. For “friend delivery,” patients would request a test from a close friend and the friend would deliver the test from a local testing clinic. For “clinic pick-up,” patients would pick up a test from a local testing clinic.

The descriptions for the support attribute (instructions only, while you test, 1 week after delivery) provided information for each attribute level on who would help patients while using an HIV self-test and provide linkage to care for a reactive result. For “instructions only,” patients would use only written and picture instructions that come with the test and use packaged information on local testing sites to receive care for a reactive result. For “while you test,” patients would use a phone test counselor (for home delivery or clinic pick-up) or a friend (for friend delivery) to provide guidance while patients use a test. The individual providing support would connect patients with reactive results to medical care. For “one week after delivery,” patients would also use a phone test counselor (for home delivery or clinic pick-up) or a friend (for friend delivery) for support, except they would receive this support one week after the test. The individual providing support would also connect patients with reactive results to medical care. The description for delivery speed specified that options described how long it would take for a patient to receive a test. The description for price specified that options described the cost of a test for patients and that test accuracy was the same for all prices.

**Fig. 1:**
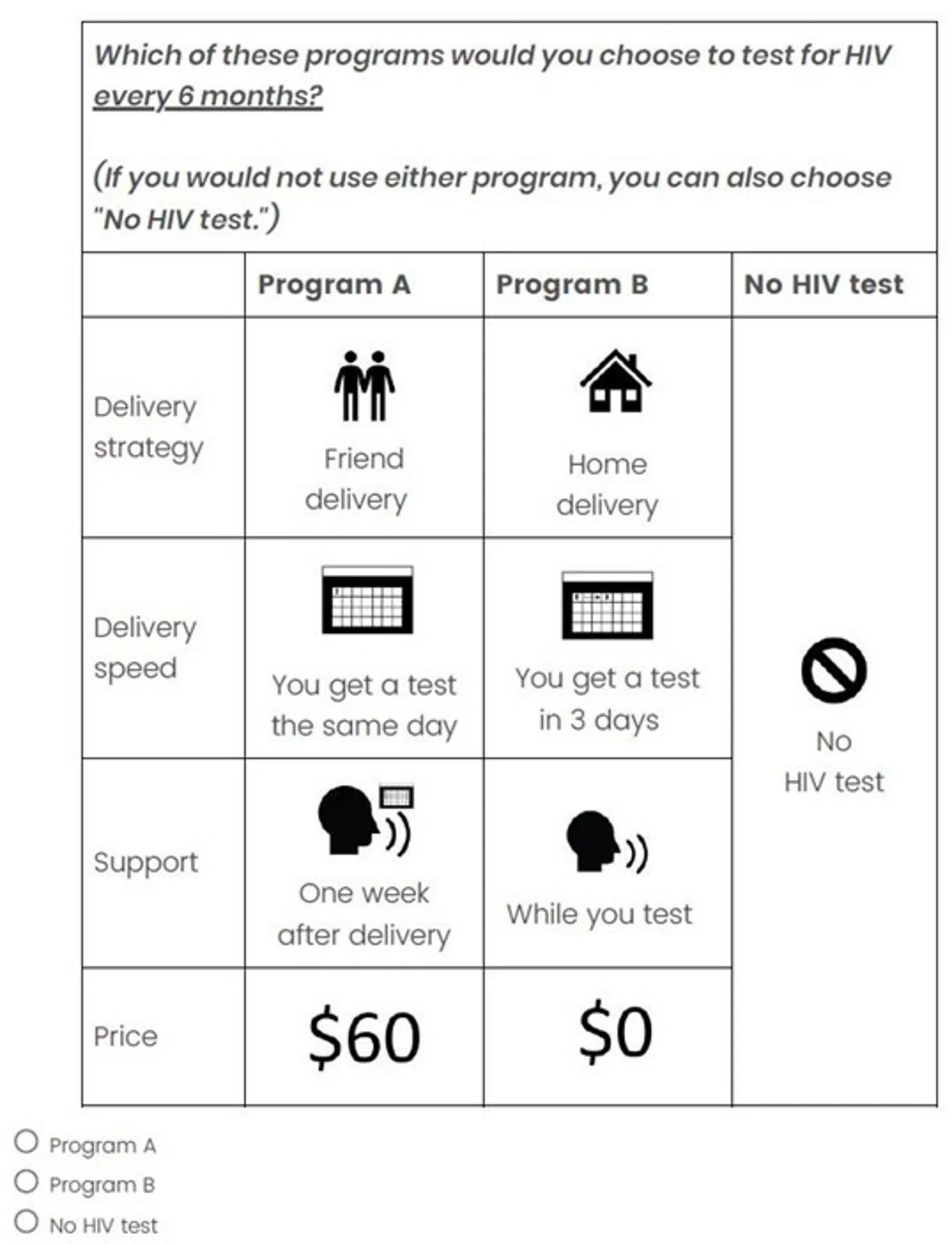
Example choice set of discrete choice experiment on HIV self-test program preferences

Figure 1 presents an example choice set. The survey randomized participants to 1 of 10 survey blocks with six unique choice sets each. Each choice set consisted of two self-test program designs (“Program A” and “Program B”) and an opt-out scenario (“No HIV test”). We asked participants to choose from possible program designs to test for HIV every six months. Using Ngene software (ChoiceMetrics, Sydney, Australia), we developed a d-efficient discrete choice experiment design with negative priors for delivery speed and price. D-efficient designs create choice sets with comparisons most likely to provide the most precise estimates by maximizing the occurrence of each potential attribute level, reducing comparisons between programs with the same attribute levels (such as between two programs with home delivery), and by incorporating priors to reduce comparisons between programs with likely dominant options (in this case, between programs with the fastest and slowest delivery times or the lowest and highest prices) [36]. We also constrained the discrete choice experiment to exclude unrealistic program designs (ex: clinic pick-up with 5-day delivery).

To contextualize the discrete choice experiment results, we also collected participant demographics, HIV testing history, and sexual risk behaviors. We determined rural status based on participant ZIP codes using data from the Health Resources and Services Administration’s Rural Health Grants Eligibility Analyzer [37].

### Analysis

We calculated proportions and means with standard deviations for participant characteristics using Stata/SE 16 (StataCorp LLC, College Station, TX). We used a random parameters logit model to estimate preferences for program attribute levels [27]. This model uses the inherent multilevel structure of discrete choice experiment data (level 1: choice set responses, level 2: individual participants) to account for individual-level heterogeneity in preferences. We used effects coding and set random parameters with normal distributions for each attribute level. A random parameters logit model requires running simulations to generate estimates [38], so we used maximum likelihood estimation with 500 Halton draws.

The resulting preference coefficients (in units of “preference weight”) and corresponding standard deviations for each attribute level are averages of estimates from all 500 draws and thus each have a corresponding standard error and p-value. Positive coefficients indicate a relative preference for that attribute level, and negative coefficients indicate a relative preference against (or dislike of) that attribute level. Standard deviations provide a measure of preference heterogeneity. A statistically significant standard deviation indicates that subgroups across the sample may have contrasting preferences for that attribute level. In addition, to understand the importance of each attribute in the participation of a self-test program, we also calculated the relative importance of each attribute by dividing the range of values of each attribute by the sum of the range of values for all attributes [27].

To test for potential differences in preference between key demographic groups that may inform the tailoring of HIV self-test program designs or pricing strategies, we ran separate random parameters logit models with interactions for race/ethnicity (non-Hispanic Black v. Hispanic), education (less than a college education v. college education or more), income (<$40,000 v. ≥$40,000), condomless anal sex partners in the past year (> 1 condomless anal sex partner v. ≤1 condomless anal sex partner), and PrEP use (no current PrEP use v. current PrEP use).

Marginal willingness to pay estimates are based on calculating the negative ratio of the coefficient for each attribute level over a continuous coefficient for price [39]. To calculate these estimates, we ran a separate random parameters logit model and dummy coded each non-price attribute with the likely simplest and least costly attribute level as the reference: clinic pickup for delivery strategy, instructions only for support, and 5-day delivery for delivery speed. We coded price as a continuous variable with a fixed parameter. We used a fixed parameter for price instead of a normally distributed random parameter because marginal willingness to pay estimations that use a normally distributed random parameter for price generate undefined estimates since a normal distribution includes zero within its bounds and ratio estimates that divide by zero are undefined [40].

We calculated log-likelihood statistics and Akaike information criteria to assess model fit. For all estimates, we reported significance levels of < 0.05 (*) and < 0.001 (**). We used NLOGIT 6 (Econometric Software Inc, Australia) for all discrete choice experiment analyses.

## Results

Recruitment resulted in 699 surveys completed between March 2023 and December 2023. Of those surveys, we excluded 382 surveys (54.46%) as likely fraudulent based on embedded eligibility checks (i.e., verifying HIV status, reCAPTCHA score), manual checks (i.e., nonsensical free response entries), and an extremely high volume of suspicious survey responses from participants with IP addresses outside the US or with no corresponding tags to our online ads indicating how they accessed our screener. These efforts resulted in 317 surveys (45.35%). However, this sample had low representation of transgender, gender nonconforming, and nonbinary individuals (*n* = 27, 8.52%). Given the unique preferences among transgender individuals for HIV testing services [41], we limited our analyses to cisgender men who have sex with men. The final analytical sample consisted of 290 surveys.

Table 1 presents characteristics of the survey sample. About half the sample was non-Hispanic Black (47.24%) and half was Hispanic (52.76%). Out of Hispanic participants in this study, 17 (11.11%) also identified as Black. Most of the sample was gay/homosexual (81.72%), and had a higher socioeconomic status, including completing a college education (67.93%) and a yearly income of $40,000 a year or more (67.93%). Notably, nearly half the sample were currently using PrEP (43.79%).

**Table 1:** Characteristics of an online sample of non-Hispanic Black and Hispanic men who have sex with men in the Southern United States, March 2023-December 2023 (*n* = 290)

Characteristics	Mean (standard deviation)
*Age (Range: 18–72)*	35.20 (10.51)
	*Count* (%)
*Race/ethnicity*	
Non-Hispanic Black	137 (47.24)
Hispanic	153 (52.76)
*Gender identity*	
Cisgender male	290 (100)
*Sexual orientation*	
Gay/homosexual	237 (81.72)
Bisexual	40 (13.79)
Straight/heterosexual	3 (1.03)
Other/not sure	10 (3.45)
*Education*	
Less than college education	92 (31.72)
College education or more	197 (67.93)
Missing	1 (0.34)
*Annual income*	
<$40,000	93 (32.07)
≥$40,000	197 (67.93)
*Preferred language*	
English	281 (96.90)
Spanish	9 (3.10)
*Rural residence*	
Non-rural	283 (97.59)
Rural	7 (2.41)
*Current health insurance*	
Yes	249 (85.86)
No	41 (14.14)
*HIV status*	
HIV negative	281 (96.90)
Unknown	9 (3.10)
*Tested for HIV in the past year*	
Yes	243 (83.79)
No	47 (16.21)
*Current HIV preexposure prophylaxis use*	
Yes	127 (43.79)
No	163 (56.21)
*Number of male condomless anal sex partners*,* past 6 months*	
≤ 1	133 (45.86)
> 1	155 (53.45)
Missing	2 (0.69)
*Sexually transmitted infection diagnosis*, past 6 months*	
Yes	33 (11.38)
No	257 (88.62)
*Ever used an OraQuick In Home HIV Test*	
Yes	94 (32.41)
No	196 (67.59)

*Includes syphilis, rectal chlamydia, and rectal gonorrhea only

**Fig. 2:**
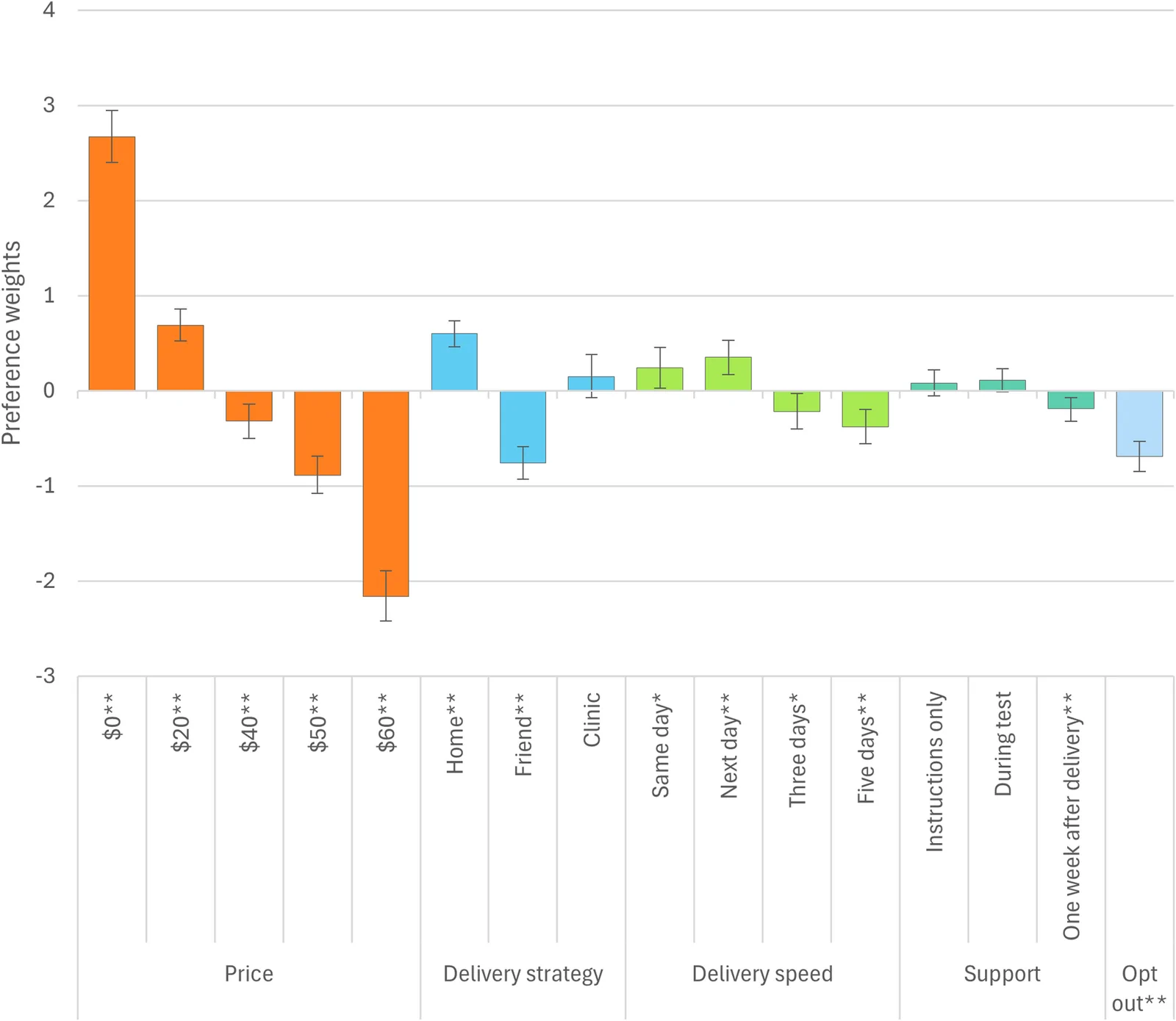
Coefficients of HIV self-test program preferences

 Table 2 presents estimates of preferences for self-test program characteristics, and Fig. 2 presents a graphic representation of these estimates. Over and above any other attribute level, participants highly preferred free self-tests (b = 2.67, SE: 0.14, *p* < 001). Participants had similar levels of preference in favor of home delivery (b = 0.60, SE: 0.07, *p*<.001), same-day delivery (b = 0.24, SE: 0.11, *p*=.029), and next-day delivery (b = 0.35, SE: 0.09, *p*<.001). Participants also had similar levels of preference against friend delivery (b=−0.76, SE: 0.09, *p*<.001), three-day (b=−0.22, SE: 0.10, *p*=.022) and five-day delivery (b=−0.38, SE: 0.09, *p* <.001). Notably, participants had a preference against support one week after delivery (b=−0.19, SE: 0.06, *p*=.002) and had no preference for support via instructions only (b = 0.08, SE: 0.07, *p*=.254) nor during the test (b = 0.11, SE: 0.06, *p*=.074). Across all choice sets, participants had a preference against opting out of an self-test program (b=−0.69, SE: 0.08, *p* <.001). In addition, price was the attribute with the highest relative importance (66.90%), followed by delivery strategy (18.84%), delivery speed (10.11%), and support (4.16%).

**Table 2:** Random parameters logit model of HIV self-test program preferences among non-Hispanic Black and Hispanic men who have sex with men in the Southern United States

Attribute	Coefficient	Standard error	*p*-value	Standard deviation	Standard error	*p*-value
*Delivery strategy*						
Home delivery	0.60	0.07	< 0.001**	1.04	0.08	< 0.001**
Friend delivery	−0.76	0.09	< 0.001**	0.88	0.12	< 0.001**
Clinic pickup	0.15	0.11	0.175	0.56	0.17	< 0.001**
*Delivery speed*						
Same day	0.24	0.11	0.029*	0.54	0.11	< 0.001**
Next day	0.35	0.09	< 0.001**	0.20	0.26	0.001*
3 days	−0.22	0.10	0.022*	0.11	0.25	0.092
5 days	−0.38	0.09	< 0.001**	0.49	0.20	< 0.001**
*Support*						
Instructions only	0.08	0.07	0.254	0.21	0.08	0.009*
During test	0.11	0.06	0.074	0.16	0.17	0.004*
1 week after delivery	−0.19	0.06	0.002*	0.14	0.19	0.019*
*Price*						
$0	2.67	0.14	< 0.001**	2.02	0.17	< 0.001**
$20	0.69	0.09	< 0.001**	0.67	0.21	< 0.001**
$40	−0.32	0.09	0.002*	0.81	0.21	< 0.001**
$50	−0.89	0.10	< 0.001**	0.88	0.21	< 0.001**
$60	−2.16	0.13	< 0.001**	1.49	0.25	< 0.001**
*Opt-out*	−0.69	0.08	< 0.001**			

*p<.05, **p<.001

Log likelihood function: −1362.72

AIC/*n* = 1.594

Table 3 presents a summary of all significant interactions. Race/ethnicity interactions indicate that non-Hispanic Black participants were more likely to choose friend delivery than Hispanic participants (b = 0.40, SE: 0.17, *p* =.020). Non-Hispanic Black participants were also less likely to choose a free test (b = 0.40, SE: 0.23, *p* <.001) but more likely to choose a $50 (b = 0.65, SE: 0.20, *p* =.024) or $60 test (b = 0.52, SE: 0.23, *p* =.024). They were also less likely to opt out of any self-test program (b=−0.49, SE: 0.17, *p* =.003). Education interactions indicate that participants with less than a college education were more likely to choose support one week after delivery (b = 0.35, SE: 0.14, *p* =.010) than participants with a college education or more. Participants with less than a college education were also less likely to choose a $40 test (b=−0.47, SE: 0.20, *p* =.019).

**Table 3:** Summary of significant interactions of random parameters logit models

Interactions	Coefficient	Standard error	*p*-value
*Attribute level x race/ethnicity interactions (ref. Hispanic)*			
Friend delivery*non-Hispanic Black	0.40	0.17	0.020*
$0*non-Hispanic Black	−0.95	0.23	< 0.001**
$50*non-Hispanic Black	0.65	0.20	0.001*
$60*non-Hispanic Black	0.52	0.23	0.024*
Opt-out*non-Hispanic Black	−0.49	0.17	0.003*
*Attribute level x education interactions (ref. college education or more)*			
One week after delivery*less than college	0.35	0.14	0.010*
$40*less than college	−0.47	0.20	0.019*
*Attribute level x condomless anal sex interactions (ref. ≤1 condomless anal sex partner*,* past year)*			
$20*>1 condomless anal sex partner	−0.36	0.18	0.040*
$40*>1 condomless anal sex partner	−0.41	0.19	0.028*
$60*>1 condomless anal sex partner	0.46	0.23	0.048*
*Attribute level x PrEP use interactions (ref. current PrEP use)*			
$60*no current PrEP use	−0.60	0.22	0.008*

*p<.05, **p<.001

Condomless anal sex interactions indicate that participants with more than one condomless anal sex partner in the last year were less likely to choose a $20 (b=−0.36, SE: 0.18, *p*=.040) or $40 (b=−0.41, SE: 0.19, *p*=.028) test than participants with one or less condomless anal sex partners but were more likely choose a $60 test (b = 0.46, SE: 0.23, *p*=.048). PrEP use interactions indicate that participants with no current PrEP use were less likely to choose a $60 test (b=−0.60, SE: 0.22, *p*=.008) than participants with current PrEP use. We found no significant interactions for income.

Table 4 presents marginal willingness to pay estimates for self-test program attributes. Participants were willing to pay $6.71 (SE: 2.29, *p*=.003) more for a test delivered via home delivery than a test accessed via clinic pickup. Participants were also willing to pay $9.10 (SE: 2.28, *p*<.001) more for a test delivered the same day and $10.96 (SE: 1.86, *p*<.001) more for a test delivered the next day compared to a test delivered in 5 days.

**Table 4:** Marginal willingness to pay estimates of HIV self-test program characteristics

Attribute	Marginal willingness to pay ($)	Standard error	*p*-value
*Delivery strategy*			
Home delivery	6.71	2.29	0.003*
Friend delivery	−16.64	2.56	< 0.001**
Clinic pickup	--		
*Support*			
Instructions only	--		
During test	1.39	1.50	0.357
1 week after delivery	−4.39	1.55	0.005*
*Delivery speed*			
Same day	9.10	2.28	< 0.001**
Next day	10.96	1.86	< 0.001**
3 days	0.39	1.92	0.838
5 days	--		

*p<.05, **p<.001

## Discussion

This online discrete choice experiment suggests that the optimal HIV self-test program among BHMSM in the Southern US is free, has same-day or next-day home delivery, and provides support via instructions only or during the test. These findings suggest that organizations and funders in the region could maximize participation in self-testing programs by aligning program designs with these preferences. Participants were also willing to pay for home delivery and faster delivery times, suggesting that patient cost-sharing could be a potential source of financial sustainability.

Our findings provide further data that over and above any other potential program characteristic, providing free tests will most effectively increase participation in self-test programs. These results coincide with previous research on price as a major barrier to accessing self-tests and that patients greatly prefer free tests [14, 42]. Moreover, these results are especially telling given that this sample consists of large proportions of participants at higher socioeconomic status, such as college education, higher income, and access to health insurance and PrEP. As such, free tests may be especially more important for participants at lower socioeconomic status who potentially have even more limited access to testing. Although it may seem evident that participants prefer tests for free, we were motivated by previous research that suggests that patients may be willing to share at least some costs of self-tests [11, 17] and the importance of cost relative to other program characteristics was not yet clear. As such, we believed it was worth exploring the potential for cost sharing given that organizations supporting the full costs of self-tests programs is likely a significant barrier to broader implementation. However, our findings that price was the most important attribute and that participants endorsed a strong preference for free tests suggest that organizations in the Southern US may achieve the greatest gains in testing from self-testing programs among BHMSM by providing tests for free or at a very low price.

In addition, our findings suggest that home delivery is also an important attribute among BHMSM for self-test programs and may yield greater participation compared to social-network delivery and clinic pickup. These results coincide with the high effectiveness of home delivery programs reported in previous randomized controlled trials [11, 15, 43] and are likely due to the high convenience and privacy of this delivery strategy [44, 45]. As such, public health departments and community organizations across the Southern US may choose to replicate successful self-test home delivery programs for BHMSM [46, 47] and grantors may choose to finance such projects.

This survey also provides empirical research on the limited importance that patients place on the delivery speed of self-test programs. Although some self-test home delivery programs operating in the Southern US likely have long delivery times, ranging from up to 2 days to 7 days after ordering [48, 49], organizations may increase participation among BHMSM if they can ensure same-day or next-day delivery. However, the complexity of faster delivery times may be a barrier to implementing such a program because organizations would have to build staffing, develop protocols, and pay for the additional costs of rapid home delivery. Given that this attribute had low relative importance compared to price and delivery strategy, organizations in the Southern US may choose to prioritize implementing free or low-cost self-test home delivery with some quality improvement efforts to reduce the delivery time of tests.

Our findings also suggest that support strategy was also a minor attribute for BHMSM and that they have a preference against follow up 1 week after delivery compared to support strategies such as package instructions alone (“instructions only”) or having a person available during testing (“while you test”). Despite the low importance BHMSM place on support, our findings are important because many organizations implementing self-test program reach out to patients several days after delivery to check test results and to offer counseling and resources [50], which may be an unappealing practice among BHMSM.

Despite the overall low relative importance that participants placed on the support strategies we offered in this survey, finding a more desirable support strategy could increase participation in self-test programs and further enable organizations in the Southern US to provide linkage to care and prevention resources. Researchers have identified a variety of more intensive unassisted support strategies that may supplement package instructions, such as video instructions or virtual guidance via a smartphone app or chatbot, as well as several different features of real-time support strategies, such as live demonstrations, phone/text contact or video support [51]. Further research can find the exact kinds of self-test support strategies that may effectively increase engagement among BHMSM.

Our interaction analyses provide further guidance on programmatic decisions for organizations. For example, despite the overall high interest in home delivery, non-Hispanic Black men who have sex with men may be more likely to participate in a program with social-network distribution than Hispanic men who have sex with men. This finding furthers previous research on social-network distribution as an effective strategy for BHMSM by showing greater acceptability of this strategy among non-Hispanic Black men who have sex with men [9, 13]. Given medical mistrust and HIV stigma within this group [52, 53], social-network distribution may be more acceptable among non-Hispanic Black men who have sex with men because it involves less contact with medical facilities than clinic pick up or greater privacy from roommates and family members than home delivery. In addition, despite an overall preference against support one week after delivery, BHMSM with less than a college education may be more likely to participate in a self-test program with this type of support. This finding may be related to lower health literacy or a greater need for health system navigation among those with lower education [54, 55]. As such, organizations that serve large populations of patients with low education may consider providing an option for support after delivery to assist with linkage to prevention and treatment services.

Although our findings suggest providing free tests could bring high participation, our results also suggest that patient cost sharing for rapid home delivery could contribute to the sustainability of self-test programs. Compared to estimates from a previous costing study of a free self-test home delivery program that likely followed standard delivery times (2–3 days) [16], the estimated $6.71 more that participants would pay for home delivery compared to clinic pickup in our study is nearly 25% of the previously reported $28 cost per test kit, and the estimated $9.10 that participants would pay for next-day delivery is already 60% of the previously reported $15 cost per kit for shipping and handling. As such, organizations may consider having patients partially fund a self-test home delivery program, and other funding sources such as grants can cover the remaining costs. Further implementation research is necessary to understand the feasibility and cost-effectiveness of charging participants for test kits.

In addition to our marginal willingness to pay analyses, our interaction analyses provide additional guidance on self-test pricing strategies for organizations. For example, interactions suggest that BHMSM with more than one condomless anal sex partner are more likely to pay a higher price for a test, which may be related to a potentially higher monetary value those with higher sexual risk behavior may place on tests. As such, organizations that serve large populations of patients with high sexual risk may further consider charging for tests. However, interactions also suggest that BHMSM with lower education are less likely to pay the retail value ($40) for tests. Given that lower education is also associated with HIV risk [56], organizations that serve large populations of patients with low socioeconomic status, especially low education levels, should avoid charging for tests. In addition, participants not currently on PrEP were less likely to pay a higher price for tests than current PrEP users, which may be due in part to higher income among current PrEP users [57]. Since clinical guidelines recommend regular testing for current PrEP users [58], our results further emphasize that organizations aiming to increase access to testing for groups with limited access to testing should avoid charging for tests.

This study is subject to limitations. First, this study has lower representation among those who test less than the recommended Centers for Disease Control and Prevention guideline of at least once a year [59], in addition to those with low-income status, low education levels, limited medical access, and limited English proficiency, which are all factors that are associated with low testing among BHMSM [60, 61]. Relatedly, this study also has low representation of participants in rural settings and transgender, gender nonconforming, and nonbinary individuals, which are also groups which face barriers to testing [62, 63]. As such, this study is lacking the preferences for self-testing programs from BHMSM who would most benefit from more strategies to access testing. Future studies should focus more intensive recruitment of those who test infrequently and those with low socioeconomic status, Spanish speakers, rural residents, and gender diverse individuals.

Second, this sample had high representation of current PrEP users. In addition to recommendations for regular laboratory HIV testing for current PrEP users, clinical guidelines do not recommend HIV self-testing for this group because antibody testing may miss acute HIV infections [58]. As such, the preferences of current PrEP users have less programmatic relevance given their greater access to highly accurate testing and low need for self-tests. However, aside from the one significant interaction showing a lower likelihood of paying a higher price for a test among those not currently on PrEP, our PrEP use interactions showed very little evidence of differences in preferences by PrEP use, suggesting that the results from our main model still largely apply to BHMSM not currently on PrEP. Moreover, given high PrEP discontinuation among BHMSM [64, 65], our results still remain relevant since some BHMSM may stop using PrEP and have a greater need for self-testing.

Third, given the high interest in free or $20 tests and that most marginal willingness to pay estimates were very low (<$10), our discrete choice experiment could have been strengthened with additional lower price attribute levels such as a $10 test. Additional lower price levels would have provided more granularity in participants’ preferences, especially when some patients may be especially price sensitive at lower price ranges [66]. However, given that the cost of running a self-test program is a primary implementation barrier [5, 6], we developed this discrete choice experiment with a focus on higher price levels as a way to potentially generate additional revenue from patients. Since this study further establishes that patients highly prefer free or low-cost tests, future discrete choice experiments with a marginal willingness to pay component can focus on patient preferences for self-tests at lower prices. Relatedly, given the very high preference for free tests, this study is potentially subject to the “zero-price” effect, in which participants in hypothetical costing studies greatly overestimate their preferences for free goods in real-world settings [67]. However, our results are in line with other studies that suggest a very high preference for free tests [11, 17].

Finally, this discrete choice experiment was limited to the attributes and attribute levels we examined and may exclude other important program characteristics. Although we designed this study based on program design options with the most uptake in self-test programs and the strongest evidence of effectiveness [19], future patient preference studies of self-test programs can include other important attributes, such as support provider characteristics (skilled, lay, friend, sexual partner) and delivery timing (ongoing subscription, batch, order as needed) [68]. Future studies can also examine other delivery strategies with high feasibility and potential uptake that have not been rigorously evaluated in trial settings, such as pharmacy pick up or vending machines [69, 70]. Finally, discrete choice experiments are stated preference exercises of hypothetical programs, so results may not fully represent real-life decision-making patterns. Despite this limitation, it is important to consider that discrete choice experiments have reasonable external validity and as such are appropriate for efforts to design new health programs [71].

## Conclusion

The results of this online discrete choice experiment can guide funders, community-based organizations and health departments in the Southern US in the implementation of self-test programs by informing them of the self-test program preferences of BHMSM. Our findings showed that these populations most preferred a free self-test program with rapid home delivery and support via instructions only or during the test. This program design has high potential to increase testing among BHMSM as a strategy to prevent the onward transmission of HIV. More implementation research is necessary to build effective implementation strategies that increase self-test programs in this region.

## Supplementary Information

Below is the link to the electronic supplementary material.

Supplementary material 1 (PDF 239.3 kb)
