# Supplementary material for: HIV Self-Test Program Preferences Among Non-Hispanic Black and Hispanic Men Who Have Sex with Men in the Southern United States: A Discrete Choice Experiment
Source: AIDS Behav. 2026 Apr 17;30(11):3405–17. doi: 10.1007/s10461-026-05125-2 (PMC13727875; doi:10.1007/s10461-026-05125-2)
Supplement: Supplementary file 1 [file 10461_2026_5125_MOESM1_ESM.pdf]

Manuscript Title: "HIV Self-Test Program Preferences among Non-Hispanic Black and Hispanic Men Who Have Sex with Men in the Southern United States: a Discrete Choice Experiment"

Journal: AIDS and Behavior

Supplementary File 1: Discrete Choice Experiment Attribute and Attribute Level Prompts

"Now we want you to choose between different HIV home-testing programs that provide this test. We want you to think about using these programs for regular health screening **(every 6 months).**

"Each program will have different options for: **delivery strategy, support, delivery speed, and price.**

"Please read the following info on these options. **This info will help you answer questions later in the survey:"**

Delivery strategy options	
Home delivery<br>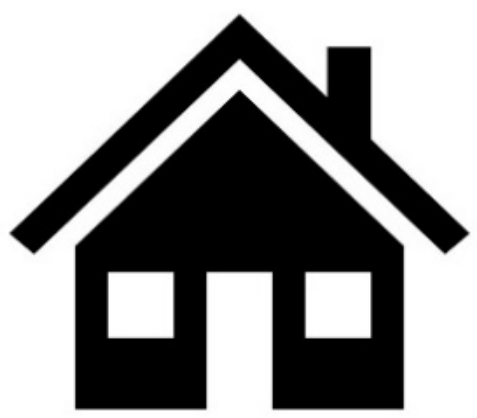	You order a test though a website of a popular local HIV testing clinic. You pay for the test online. The clinic mails a test to your home.
Friend delivery<br>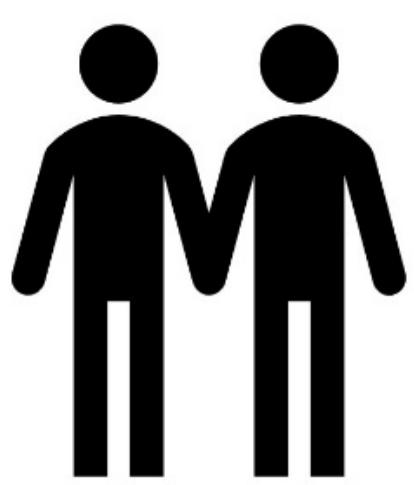	You have a friend who signed up with a popular local HIV testing clinic to deliver tests. The clinic trained your friend on how to use the test and how to help if you have a positive result. The clinic paid your friend for the training and to deliver tests.<br><br>You ask this friend for a test. You pay your friend when you get the test, and your friend gives that money to the clinic. The clinic never asks your name or contact info.
Clinic pick-up<br>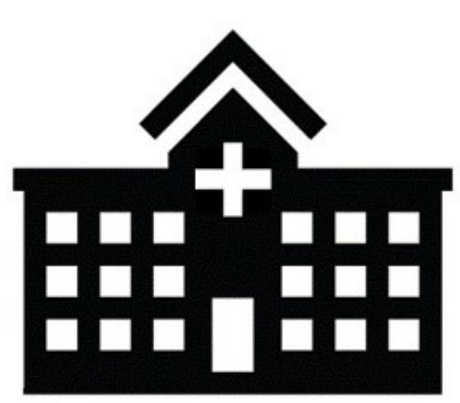	You go to a popular local HIV testing clinic for a test. You pay the clinic for the test. You take the test home to use.

Support options	
Instructions only<br>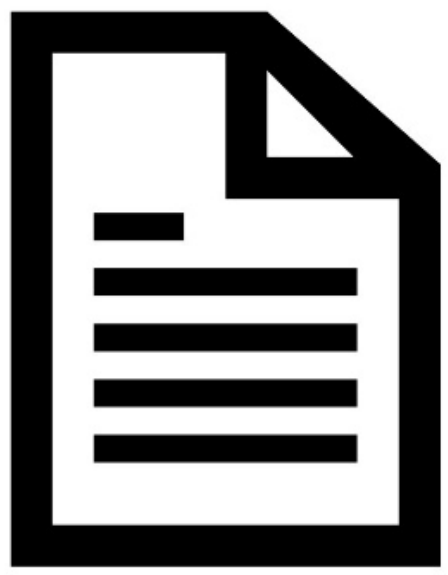	The test comes with written and picture instructions. It includes info for local HIV testing. If you have a positive result, you can use this info to access lab testing to confirm your results and get medical care.
While you test<br>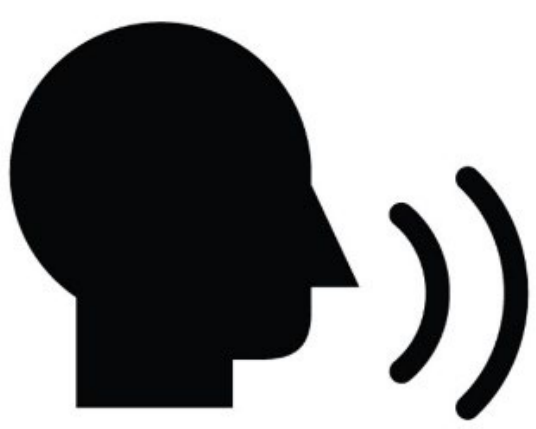	A **phone HIV test counselor** (for home delivery or clinic pick-up) or **your friend** (for friend delivery) helps you use the test. If you have a positive result, they can connect you to lab testing to confirm your results and get medical care.
One week after delivery<br>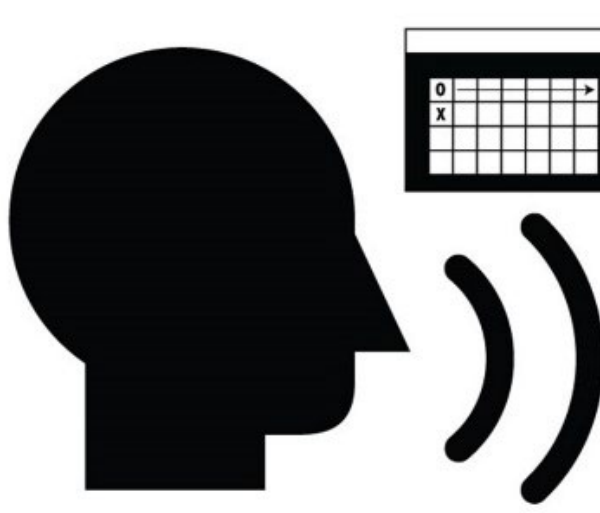	A **phone HIV test counselor** (for home delivery or clinic pick-up) or **your friend** (for friend delivery) contacts you one week after you get the test. If you have a positive result, they can connect you to lab testing to confirm your results and get medical care.

Delivery speed options	
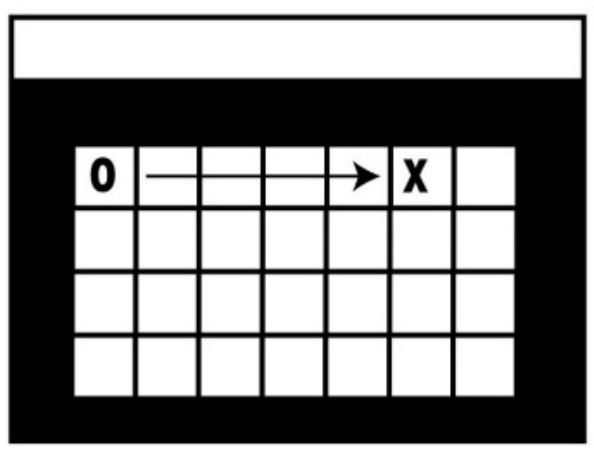	How long it takes to get a test. The test is the same for all delivery times. The example image to the left is for a delivery speed of five days.

Price options	
$40	The cost of a test to you. The test is the same for all prices. The example image to the left is for a program with a $40 test.
